# SMARTHealth India: Development and Field Evaluation of a Mobile Clinical Decision Support System for Cardiovascular Diseases in Rural India

**DOI:** 10.2196/mhealth.3568

**Published:** 2014-12-08

**Authors:** Devarsetty Praveen, Anushka Patel, Arvind Raghu, Gari D Clifford, Pallab K Maulik, Ameer Mohammad Abdul, Kishor Mogulluru, Lionel Tarassenko, Stephen MacMahon, David Peiris

**Affiliations:** ^1^The George Institute for Global Health, IndiaHyderabadIndia; ^2^Sydney Medical SchoolUniversity of SydneySydneyAustralia; ^3^The George Institute for Global HealthUniversity of SydneySydneyAustralia; ^4^Institute of Biomedical EngineeringDepartment of Engineering ScienceUniversity of OxfordOxfordUnited Kingdom; ^5^Department of Biomedical InformaticsEmory University School of MedicineEmory UniversitySouth AtlantaGeorgia; ^6^The George Institute for Global HealthOxford UniversityOxfordUnited Kingdom

**Keywords:** clinical decision support systems, mobile health, developing countries, community health worker, primary health care, cardiovascular disease

## Abstract

**Background:**

Cardiovascular disease (CVD) is the major cause of premature death and disability in India and yet few people at risk of CVD are able to access best practice health care. Mobile health (mHealth) is a promising solution, but very few mHealth interventions have been subjected to robust evaluation in India.

**Objective:**

The objectives were to develop a multifaceted, mobile clinical decision support system (CDSS) for CVD management and evaluate it for use by public nonphysician health care workers (NPHWs) and physicians in a rural Indian setting.

**Methods:**

Plain language clinical rules were developed based on standard guidelines and programmed into a computer tablet app. The algorithm was validated and field-tested in 11 villages in Andhra Pradesh, involving 11 NPHWs and 3 primary health center (PHC) physicians. A mixed method evaluation was conducted comprising clinical and survey data and in-depth patient and staff interviews to understand barriers and enablers to the use of the system. Then this was thematically analyzed using NVivo 10.

**Results:**

During validation of the algorithm, there was an initial agreement for 70% of the 42 calculated variables between the CDSS and SPSS software outputs. Discrepancies were identified and amendments were made until perfect agreement was achieved. During field testing, NPHWs and PHC physicians used the CDSS to screen 227 and 65 adults, respectively. The NPHWs identified 39% (88/227) of patients for referral with 78% (69/88) of these having a definite indication for blood pressure (BP)-lowering medication. However, only 35% (24/69) attended a clinic within 1 month of referral, with 42% (10/24) of these reporting continuing medications at 3-month follow-up. Physicians identified and recommended 17% (11/65) of patients for BP-lowering medications. Qualitative interviews identified 3 interrelated interview themes: (1) the CDSS had potential to change prevailing health care models, (2) task-shifting to NPHWs was the central driver of change, and (3) despite high acceptability by end users, actual transformation was substantially limited by system-level barriers such as patient access to doctors and medicines.

**Conclusions:**

A tablet-based CDSS implemented within primary health care systems has the potential to help improve CVD outcomes in India. However, system-level barriers to accessing medical care limit its full impact. These barriers need to be actively addressed for clinical innovations to be successful.

**Trial Registration:**

Clinical Trials Registry of India: CTRI/2013/06/003753; http://ctri.nic.in/Clinicaltrials/showallp.php?mid1=6259&EncHid=51761.70513&userName=CTRI/2013/06/003753 (Archived by WebCite at http://www.webcitation.org/6UBDlrEuq).

## Introduction

Cardiovascular diseases (CVD) are the major cause of premature death and disability worldwide and are rapidly rising in many low- and middle-income countries (LMICs) [1-3]. In India, CVD risk factor levels are high in rural populations, which currently constitute 70% of the total population, and CVD is now the leading cause of adult deaths in many rural communities [4,5]. Despite the availability of evidence-based guidelines for the prevention of CVD, the use of simple, affordable, preventive treatments (eg, smoking cessation strategies, aspirin, low-cost statins, ACE-inhibitors, and beta-blockers) is very low in these communities [6]. Even the use of secondary preventive drugs is low among people with known coronary heart disease (CHD) or stroke [7]. Multiple barriers exist at different levels of the health systems, including lack of health care facilities, limited access to health care providers, and high out-of-pocket costs for consumers [8].

Several systematic reviews have consistently shown that clinical decision support systems (CDSS) are able to improve effectiveness of care [9-15]; however, the vast majority of evaluations have been conducted in high-income countries. Given the vastly different health systems and infrastructure, the effectiveness of CDSS in LMIC settings is unclear [16]. As a result of infrastructure limitations for fixed-line information technology solutions, implementation of CDSS in these settings could be enhanced by building them into accessible and affordable mobile device-based platforms. A few recent reviews in LMICs concluded, however, that the current evidence for their effectiveness is limited mostly to isolated interventions with intermediary outcomes, such as cost savings, improved data quality, monitoring and supervision, and improved quality of care [17-22]. One of the reviews advocated mobile health (mHealth) solutions that are integrated within existing health system structures and recommended the use of end-to-end solutions ranging from point-of-care support through to high-level system elements such as shared electronic records and processes to manage drug supply chain [17].

India has a 3-tier health care system, with a subcenter at the village level, primary health center (PHC) at the mandal level (a group of approximately 10 villages), and a community health center (for every 4 PHCs) at the district level. The PHC, often led by 1 doctor, is expected to provide comprehensive primary health care for up to 30,000 residents. This leads to massive unmet demand, placing considerable strain on PHC resources and consequently on the quality of care provided. In this context, there is an urgent need for different workforce strategies. One promising solution is to expand the capacity of nonphysician health care workers (NPHWs) by training and provision of appropriate tools [23]. Within each village, 1 local female resident, with approximately a grade 10-level education, is appointed as an accredited social health activist (ASHA) for every 1000 residents. ASHAs receive performance-based remuneration under India’s National Rural Health Mission program. On average, they work for 2-3 hours each day, with a primary focus on maternal and child health. Their services are provided primarily through outreach village household visits, which provide an ideal environment for additional opportunistic health screening activities and reaching sectors of the community that may not readily access the PHC. A trial conducted in this region demonstrated that a simple algorithm administered in the community by NPHWs increased the identification of people at high risk of cardiovascular events (ie, individuals with a history of heart attack, stroke, or angina) who would be eligible for proven preventive drug therapies [24]. This suggests that this workforce can be trained to effectively identify people at high risk and refer them appropriately for medical care.

In this paper, we describe an innovative strategy called Systematic Medical Appraisal Referral and Treatment in India (SMARTHealth India) that comprises (1) a mobile device–based CDSS for CVD risk management, (2) task-shifting traditional physician roles to NPHWs, and (3) integration of the overall system within government PHC infrastructure in rural India. Our objectives were (1) to develop a valid CVD risk assessment and management algorithm based on best practice national and international recommendations, with a focus on blood pressure (BP) management and (2) to assess the utility, preliminary effectiveness, and acceptability of the system among community members, NPHWs, and PHC physicians.

## Methods

### Ethics Statement

The study was approved by the ethics committee of the Centre for Chronic Disease and Control, New Delhi, and the University of Sydney, New South Wales, Sydney. Informed, written consent was obtained from all participants contributing data in the study. This is a registered study (CTRI/2013/06/003753).

### Clinical Decision Support System Development

Plain language clinical rules were developed based on a synthesis of recommendations from Indian and international screening and management CVD guidelines. World Health Organization (WHO)/International Society of Hypertension (ISH) risk charts calibrated for use in India were used for assessment of 10-year risk of a fatal or nonfatal cardiovascular event (eg, myocardial infarction or stroke) [25]. Only those conditions for which information could be collected in the primary care settings were programmed into the algorithm. Overall, this resulted in 28 inputs and 42 calculated variables in the algorithm (Textbox 1).

**Variables and conditions for use of WHO/ISH risk charts in this algorithm.**

Variables used in the WHO/ISH risk chart:

- Age
- Sex
- Smoking status
- Presence or absence of diabetes
- Systolic blood pressure
- Total cholesterol

Individuals at high cardiovascular risk include the following:

- Presence of established cardiovascular disease
- Without established cardiovascular disease and:
  - Systolic blood pressure ≥160 mm Hg
  - Diastolic blood pressure ≥100 mm Hg
  - Total cholesterol (TC) ≥320 mg/dL
  - Low-density lipoprotein cholesterol (LDL-C) ≥240 mg/dL
  - TC/High-density lipoprotein cholesterol (HDL-C) >8

Risk is considered to be underestimated in the presence of any of the following conditions:

- Taking antihypertensive therapy
- Obesity (including central obesity)
- Family history of premature coronary heart disease (CHD) or stroke in first-degree relative (male <55 years, female <65 years)
- Raised triglyceride level (>2.0 mmol/L or 180 mg/dL)
- Low HDL cholesterol level (<1 mmol/L or 40 mg/dL in males, <1.3 mmol/L or 50 mg/dL in females)
- Fasting dysglycemia
- Raised resting heart rate (heart rate >100 beats per minute)

If age is between 18 and 40 years, risk is determined at age imputed at 40 years; if age is ≥79 years, risk is determined at age imputed at 79 years.

Depending on the availability of cholesterol testing, low and high information risk equations were programmed with the algorithm automatically defaulting to the appropriate equation depending on the data available. Once risk was assessed, management recommendations were derived based on Indian CVD-related guidelines (Textbox 2).

These plain language rules were then built as a java-based application into a mobile tablet computer using the Android 4.1 operating system. Both English and local language (Telugu) versions were developed. Based on ASHA literacy levels, a 7-inch screen size was chosen to maximize utility but still maintain the portability afforded by smaller mobile phone–size screens.

**CVD risk management outputs and targets programmed in the algorithm.**

BP-lowering medication indications:

- Established CVD
- BP ≥160/100 mm Hg
- BP ≥140/90 mm Hg and 10-year CVD risk >20%
- BP ≥130/80 mm Hg and 10 year CVD risk >30%
- TC ≥320 mg/dL or LDL-C≥240 mg/dL or TC/HDL-C>8

Lipid-lowering medication (statins) indications:

- Established CVD
- Established diabetes
  - Previous diagnosis of diabetes
  - Fasting blood glucose ≥126 mg/dL
  - Random blood glucose ≥200 mg/dL
- TC ≥ 320 mg/dL or LDL-C≥240 mg/dL or TC/HDL-C>8
- BP ≥160/100 mm Hg
- 10-year CVD risk >30%
- Age ≥40 years and 10-year CVD risk >20% and any 1 of the following:
  - TC ≥200 mg/dL
  - LDL-C≥120 mg/dL

Antiplatelet medication indications:

- Established atherosclerotic CVD
- TC ≥320 mg/dL or LDL-C≥240 mg/dL or TC/HDL-C>8
- BP ≥160/100 mm Hg
- 10-year CVD risk >30%

Target treatment levels for BP:

- BP <130/80 mm Hg for those with any of the following:
  - Established CVD
  - Established diabetes
  - TC ≥320 mg/dL or LDL-C≥240 mg/dL or TC/HDL-C>8
- BP <140/90 mm Hg for all others

Target treatment levels for lipids:

- TC <160 mg/dL, LDL-C<80 mg/dL, and HDL-C>45 mg/dL for those with established CVD
- TC <180 mg/dL, LDL-C<100 mg/dL, and HDL-C>45 mg/dL for those with diabetes
- TC <200 mg/dL, LDL-C<120 mg/dL, and HDL-C>40 mg/dL for all others

**Figure 1:**
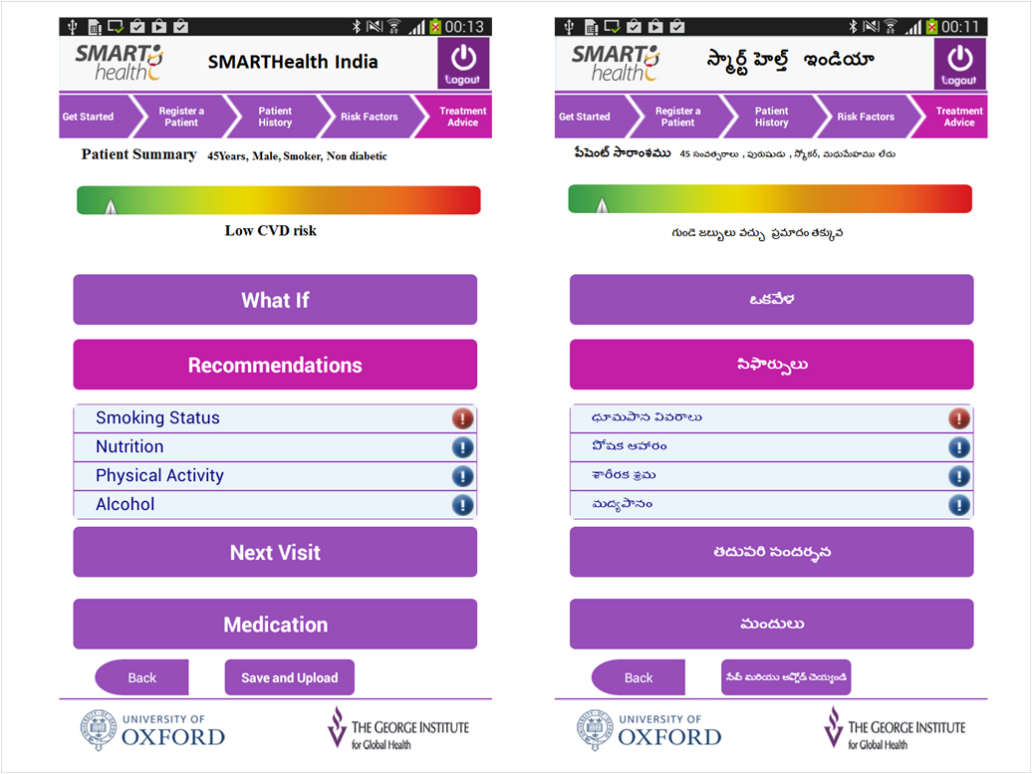
Treatment advice screen of the app in English and Telugu (local language).

The app takes the user through a 4-step process (patient registration, past medical history and medications, risk factor measurements, and treatment advice). For BP measurement, the ASHA/physician used a Bluetooth-enabled automatic monitor to wirelessly upload readings to the computer tablet. Blood glucose, cholesterol (if available), height, and weight are manually entered. The treatment advice page provides the 10-year CVD risk of the participant, lifestyle and referral recommendations for the ASHA, and medication recommendations for the physicians (Figure 1).

Data are stored locally on the tablet and are securely uploaded to a server hosted at the coordinating research institute using Open Medical Record System (OpenMRS) version 1.9. Data uploads occur asynchronously whenever there is an adequate network connection available.

### Validation Testing of the Clinical Decision Support System Tool

The algorithm was validated using a 2-step process following methods used in previous studies [26].

Step 1 was a comparison with an independently coded program. Each of the 42 calculated variables in the algorithm was tested using deidentified data from 200 individuals involved in the Andhra Pradesh Rural Health Initiative [24]. A research fellow independently coded the plain language algorithm rules into SPSS statistical software package [27] and the outputs generated from the CDSS and SPPS programs were compared. In an iterative process, outputs were examined, programming modifications were made, and variables were retested to ensure they were programmed correctly.

Step 2 was physician-based validation. A random sample of 100 patient cases was constructed again using deidentified data from the Andhra Pradesh Rural Health Initiative [24]. A physician, who was not involved in the algorithm development, independently reviewed these cases. She assessed 10-year CVD risk for each case using paper WHO/ISH risk charts and manually reviewed the Indian guidelines to provide management advice. Agreement was then assessed between the CDSS and the physician’s recommendations.

Once all adjustments were made, a larger deidentified primary health care dataset comprising 1000 patients was then tested in both the CDSS and SPSS programs. Agreement between the outputs obtained from the 2 programs was again assessed.

### Field Testing

The CDSS was field-tested in 11 villages and 3 PHCs in the West Godavari district of Andhra Pradesh, India. A 5-day and 1-day training course was provided for ASHAs and physicians, respectively. Following this, ASHAs conducted village-based opportunistic CVD risk assessments for a minimum of 20 households per village over a 1-month period, while doctors did the same in the PHC setting. Anyone identified by the ASHA to be at high CVD risk was provided with a referral card to attend the local PHC.

### Evaluation and Analysis

A mixed methods evaluation was conducted. Quantitative and qualitative components were equally weighted and combined simultaneously to obtain an understanding of the likely effectiveness, acceptability, and feasibility of the CDSS. Deidentified clinical data and “loggers” that recorded usage data from each page in the tablet were collected. Although the study was not designed for the purpose of evaluating effectiveness, clinical data were analyzed to assess the number of patients identified as high risk provided with appropriate management by the doctor, with a particular focus on BP management. A follow-up survey was also conducted for the high-risk patients referred to the PHC to assess the numbers who attended and the numbers who initiated treatment. Quantitative analyses were performed using SPSS version 21 (IBM Corp, Armonk, NY, USA).

**Figure 2:**
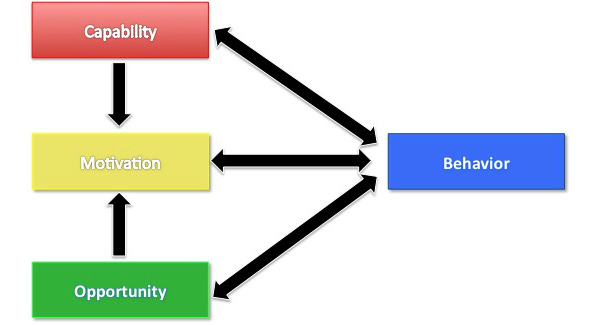
The COM-B framework for understanding behavior.

At the end of the study, all physicians and ASHAs participated in an in-depth interview evaluation and selected community members participated in 4 village-based focus group discussions (separate male and female groups). Interviews were semistructured and conducted by a researcher experienced in these field settings who was proficient in English and Telugu. The COM-B theory of behavior change (Figure 2) was used to design and analyze the interview data [28]. This theory looks at 3 interrelated domains which are linked to behavior change: *capability* refers to a physical and psychological capacity to engage in an activity, *motivation* refers to automated and reflective brain processes that energize and direct behavior, and *opportunity* refers to all the physical and social factors that lie outside of the control of an individual that influence change [28].

A systems lens to the behavior change was considered and the manner in which intervention influenced the behavior of several different actors (patients, ASHAs, and doctors) to improve CVD risk detection and management was evaluated. Interviews covered the following domains: (1) staff roles and responsibilities; (2) patient, ASHA, and doctor satisfaction with using the tablet; (3) staff knowledge and skills; and (4) impact of CDSS on usual work routines. Interviews were professionally transcribed, translated to English, and thematic content analysis performed [29]. Transcripts were open-coded to key thematic domains using NVivo 10 (QSR International, Melbourne, Australia) and these were interpreted with reference to the COM-B model. The broader research team met regularly to corroborate these themes and collective knowledge on health systems in India and elsewhere was drawn upon to better understand the impact of the intervention.

## Results

### Validation of the Tool

For the initial 200 patient dataset, there was initial agreement between the CDSS output and output from the SPSS program for 29 of 42 calculated variables (69%). The causes for the discrepancies were primarily due to misinterpretation of the plain language rules by the software engineer, some minor programming errors in both the CDSS and SPSS versions, and incoherent logic when dealing with missing values. These errors were rectified and the algorithm was then retested resulting in 100% agreement.

For the physician review of 100 cases, there was perfect agreement between the physician-calculated 10-year CVD risk and the CDSS output. For management recommendations, initially there were 4 cases of disagreement which were chiefly due to a difference of opinion for treatment of CVD patients with normal BP or cholesterol levels. Minor adjustments were subsequently made to the algorithm. Once all final adjustments were made, the algorithm was revalidated using the 1000-patient dataset and perfect agreement was obtained.

### Field Testing: Quantitative Evaluation

The recruited ASHAs were diverse in age, schooling, work experience, and English literacy (Table 1). The 3 doctors were similarly diverse in age (29-44 years), years of clinical experience (4-20 years), and time working in the PHC (2-11 years).

**Table 1:** Characteristics of health workers (ASHAs) in the study (N=11).

Variable		n (%)
**Age group**		
	20-29 years	5 (45)
	30-39 years	5 (45)
	40-49 years	1 (9)
**Gender**		
	Female	11 (100)
**Schooling**		
	Primary (completed grade 5)	5 (45)
	Secondary (completed grade 10)	6 (55)
	Higher (completed grade 12)	—
**English communication**		
	Read and write	8 (73)
	Cannot read or write	3 (27)
**Working as ASHA** ^a^		
	2 or less years	2 (20)
	2 to 4 years	4 (40)
	4 to 6 years	0 (-)
	6 to 8 years	4 (40)
**Average working hours per day** ^a^		
	2 or less hours	7 (70)
	2 to 4 hours	2 (20)
	4 to 6 hours	1 (10)
**Use of cell phone**		
	Personal	4 (36)
	Shared with other family members	7 (64)

^a^ One missing response.

ASHAs and physicians opportunistically used the CDSS to assess 227 and 65 individuals, respectively. All staff achieved the minimum screening targets except for 1 ASHA whose tablet was damaged during the study period. Patient characteristics are highlighted in Table 2. ASHAs tended to screen a larger proportion of female patients compared with the doctors; conversely, the doctors tended to screen a larger proportion of patients who were already taking BP-lowering therapy when compared with the ASHAs. A much higher than expected proportion of participants screened by ASHAs reported a history of peripheral vascular disease (PVD) suggesting problems with interpretation of this question on the tablet device. On further review of this issue, it became clear that some ASHAs were assigning this diagnosis if the patient reported any leg pain rather than a doctor-confirmed diagnosis. Thirteen participants were classified at high CVD risk on this basis alone.

**Table 2:** Cardiovascular risk factor profile for participants screened by ASHAs (n=227) and doctors (n=65).

Variable		Screened by ASHAs (n=227)	Screened by doctors (n=65)	*P* value
Age (years), mean (SD)		51.4 (13.1)	55.3 (11.6)	.03
Female, n (%)		152 (67)	24 (37)	<.001
Current smoker, n (%)		30 (13)	23 (35)	<.001
**Past history, n (%)**				
	Angina/heart attack	36 (16)	7 (11)	.43
	Stroke	2 (1)	2 (3)	.22
	Peripheral vascular disease	22 (10)	1 (2)	.03
	Diabetes	31 (14)	13 (20)	.24
**Medication history, n (%)**				
	Blood pressure lowering	44 (19)	29 (45)	<.001
	Lipid lowering	1 (0)	2 (3)	.13
	Antiplatelet	6 (3)	2 (3)	>.99
Nondiabetics with an elevated capillary blood glucose,^a^ n (%)		37 (16)	7 (11)	.33
SBP (mm Hg), mean (SD)		129 (22)	131(17)	.48
DBP (mm Hg), mean (SD)		80(12)	83(12)	.08
Weight (kg), mean (SD)		57.9(16.3)	62.3(14.7)	.05
BMI (kg/m^2^), mean (SD)		24.2(4.6)	24.1(4.8)	.88
**10-year adjusted cardiovascular risk, n (%)**				
	<10% risk	122 (54)	36 (55)	.89
	10-20% risk	17 (7)	8 (12)	.22
	20-30% risk	8 (4)	2 (3)	>.99
	30-40% risk	6 (3)	1 (2)	>.99
	>40% risk	2 (1)	2 (3)	.21
	Clinically high risk^b^	20 (9)	7 (11)	.63
	CVD	45 (20)	9 (14)	.36
	CVD and CHR	7 (3)	—	.35

^a^ Elevated capillary blood glucose defined as random glucose ≥200 mg/dL or fasting glucose ≥126 mg/dL.

^b^ Defined as presence of raised blood pressure (>160/100 mm Hg), without established CVD.

**Figure 3:**
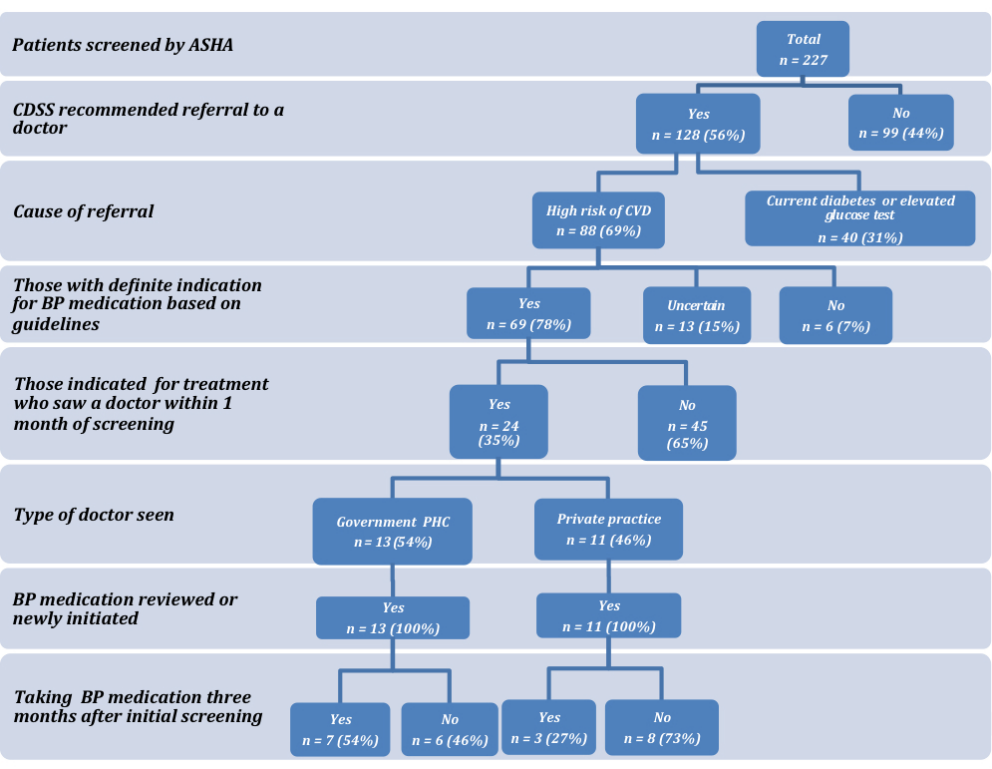
Assessment and management pathway for patients screened by the ASHA (n=227).

For those patients referred to a physician, approximately one-half visited the PHC doctor as opposed to a private practitioner. Two management gaps were identified (Figure 3). Only 35% (24/69) who had a clear indication for initiating BP-lowering medication actually visited a doctor within 1 month of screening. Although all 24 patients who accessed either a government or private doctor were recommended BP-lowering medicines, only 42% (10/24) reported still taking medication at 3-month follow-up. The majority (70%, 7/10) of those that were adherent at 3 months had visited a government doctor. Other CVD medications potentially indicated, such as antiplatelet and lipid-lowering medications, were not prescribed to any patients despite indications for 1 of these in 24 patients. Because the decision support tool only provided treatment recommendations for CVD risk management, the only advice provided for those with elevated glucose (n=31) or those with a past history of diabetes (n=9) who were not at elevated CVD risk was that they see the doctor for further testing. We did not capture any further information on the management practices of these patients. Patients diagnosed with PVD by the ASHAs but lacking any other data to suggest high CVD risk (n=13) were excluded from further analyses.

**Figure 4:**
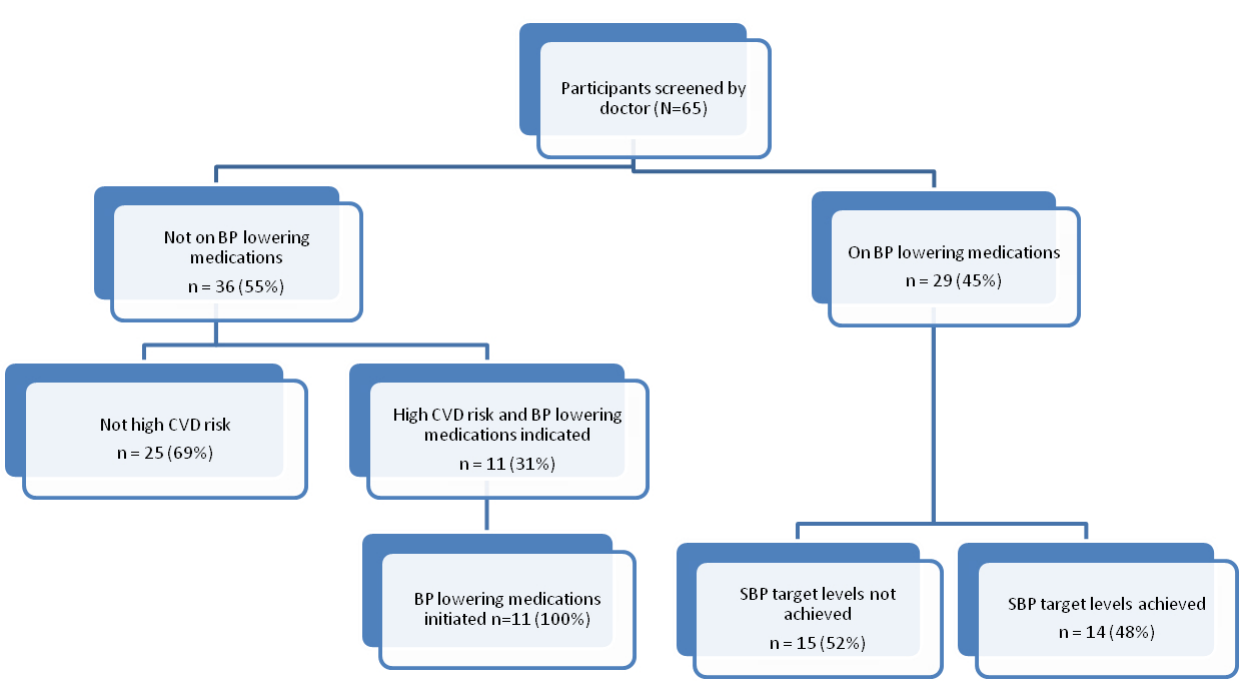
Blood pressure management in participants screened by the physician (n=65).

Of the patients assessed by doctors (Figure 4), 45% (29/65) were already taking BP-lowering medications but target levels were reached in only 48% (14/29). Among those not on medications, 31% (11/36) were recommended for treatment by the CDSS. The physician commenced all these patients (n=11) on BP-lowering treatment.

### Field Testing: Qualitative Evaluation

**Figure 5:**
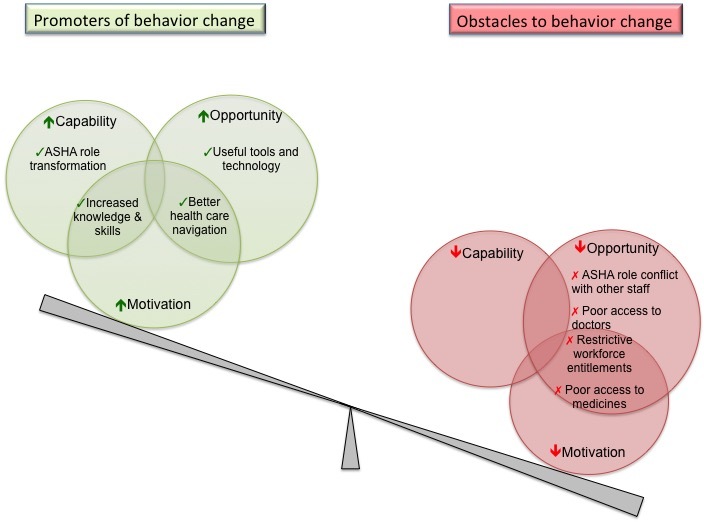
Illustration of the interview themes in context of the COM-B model.

All physicians and ASHAs participated in interviews and 4 community focus groups were conducted (2 male and 2 female groups). Interviews lasted for approximately 60 to 90 minutes. Three interrelated interview themes emerged: (1) the intervention strategy had potential to transform prevailing health care models, (2) task-shifting of CVD screening to the ASHA was the central driver of change, and (3) despite high acceptability, *actual* transformation was substantially limited by system-level barriers such as access to doctors and medicines. These themes are expanded subsequently in the context of the COM-B model. Figure 5 is a diagrammatic representation of themes identified that both enhanced (green circles) and inhibited (red circles) behavior change. These are further broken down into the 3 domain areas of capability, opportunity, and motivation. Where a theme is in the overlapping areas, it means that this theme influenced 2 domains.

### Potential to Change Prevailing Health Care Models

#### Acquisition of New Knowledge and Skills: Increased Capability and Motivation

Although ASHAs had some knowledge about CVD, few had received formal training in this area. Mostly, their role had been confined to assisting other staff in collecting BP information; however, in this setting ASHAs became proficient in not only performing risk factor measurements, but also in interpretation of the results:

Earlier, I just used to go and measure BP, but with this tablet, I came to know what was a normal reading and how the actual reading differs from normal readings.

ASHA 1, 31 years

Several ASHAs considered that the training and support provided in this project improved their knowledge and that the tablet itself offered a novel mechanism for dissemination of this knowledge to the community. The doctors found that the training and support enhanced their awareness of guideline recommendations for lifestyle and medication management of cardiovascular risk. They described how this system increased their confidence in providing treatment advice based on the overall risk score.

#### Tools and Technology: Increased Opportunity

Overall, patients and providers received the technical aspects of the tablet and the CDSS positively. Several ASHAs described initially feeling anxious and some were skeptical of their ability to use electronic devices. However, these sentiments rapidly dissipated and by the third day of the initial training course, the majority were using the tablet device confidently:

First, we were afraid that there was the need to handle computers and touch screens, but later after training, we were able to understand it. After we did 1 or 2 tests, it became easy, and we can do it better now.

ASHA 11, 29 years

Over the course of the field implementation, this confidence noticeably grew and most ASHAs described how the tablet was able to facilitate skills expansion into areas they previously would not have considered possible:

For people at my level to inform others about their health conditions is really a fortunate thing. Usually for these things, only specialists can talk about them. But with this tablet we are able to tell them what are the present levels and what would be future levels (of risk), and to what extent one needs to take care...

ASHA 11, 29 years

For the doctors, the tablet appeared to enhance workflow and was viewed as a time-saver. It also increased their confidence in communicating with patients. One doctor also described how the patient perceptions of the value of the tool enhanced the quality of the health care interaction:

It’s wonderful. I got better results than I expected...If patients see the risk bar, they understand very well that they have a high risk of CVD...We gained knowledge from this percentage display too...This is 100% beneficial to the doctor.

DOCTOR 2, 48 years

There were detailed accounts from all those interviewed on the optimal ways to communicate risk. Of all the mechanisms used in the tablet to express risk (color, percentage score, and “what if” scenarios to show risk changes), there was a strong preference for the use of color, particularly for a population with low levels of literacy:

If it (the arrow) is turned...if it becomes red, then it is danger...Red signs are kept on the road as danger...

Male community focus group 1

Although overall the use of a tablet was favorably received, a number of technical limitations were also encountered which would affect larger scale use. Hardware limitations included limited battery life, screen damage, challenges with Bluetooth pairing, and inaccurate rendering of the local language font (Telugu) in the tablet. Although continuous network connectivity was not necessary, there were some villages with very limited connectivity that led to prolonged delays in the transmission of the data to the central server.

#### Removal of Barriers to Navigating Health Care: Increase Opportunity and Motivation

A major transformative element of the strategy was its ability to reduce access barriers. The community positively received village-based screening:

We do not have transportation facility, no autos—it is like living in a jungle, and so we are happy that she (ASHA) is around. She is not only taking care of fevers...but also testing our sugar and blood pressure...

Male community focus group 2

Most ASHAs highlighted ease of access to households. Only 1 ASHA described isolated pockets in her village that were not accessible because the residents came from a higher caste. Similarly, most community participants who were recommended for referral to the doctor appeared very receptive. Several ASHAs described a duty of care to reach men, which represents a substantial departure from the current maternal and child health–focused role. However, some ASHAs found it challenging to screen male community members due to long working days and were reticent about doing after-hours screening. Despite these reservations, men highlighted that accessibility after hours was considered a highly valued service. Most men visited the ASHA’s residence during the evenings and did not express any inhibitions with this.

### ASHA Task-Shifting Role as the Central Driver of Change

#### Role Transformation: Increased Capacity

ASHAs described feeling welcomed by the community in their new role and described a sense of respect at being given additional responsibilities in this project. Community participants also expressed satisfaction with the convenience and reduced costs of village-based screening by the ASHA:

We know there are expenses involved with these tests. So, if it is done here, there is no expenditure for us...I never thought I had sugar problems, but when I came to her (ASHA), she tested me and found that I had sugar up to 360...

Female community focus group 2

Importantly, community members expressed high levels of confidence in the ASHAs ability to perform this role:

Once I got examined by a nurse in town and she did an excellent job. And here she (ASHA) examines me even better than that nurse...

Male focus group 1

This role extended from screening to also engaging patients in management advice for lifestyle actions. One focus group participant commented on his decision to cease tobacco use and alcohol consumption following advice from the ASHA:

I have been drinking alcohol and used to fall down and I stopped it now because I was told by my (ASHA)...Most of us have now stopped smoking bidi, cigarette...

Male focus group 1

One doctor also described how the “frontline” role of the ASHA placed them in an ideal position to motivate patients to make lifestyle changes and for treatment monitoring:

In my opinion, ASHAs can motivate lifestyle changes. If she is a good working ASHA, she can also monitor whether they are taking (medicines) or not...

DOCTOR3, 29 years

All physicians interviewed viewed the system of ASHAs referring patients to the PHC for medication as being a highly useful strategy to maximize their limited resources. One physician used the project to look at systematically incorporating the expanded ASHA role into PHC activities. A key part of this was a continuous reporting mechanism whereby he monitored the duties of his ASHAs in screening and following patients for regular medications.

#### Role Conflict: Diminished Opportunity

Although there was a high level of support for these new roles played by the ASHAs, there was potential for unintended consequences. Although motivating patients to take lifestyle action was well received, a few ASHAs expressed apprehension in communicating the CVD risk score. Disclosure of such information could be perceived as being too sensitive and, therefore, was considered to be the responsibility of the doctor or someone more qualified:

...I felt that if I tell patients (of their risk score) they may be afraid. I will give them a referral card and tell them to go to the doctor...the doctor has to tell...

ASHA 2, 30 years

At the core of this issue was not the communication of risk per se, but concerns about the lack of follow-up support if someone was identified as high risk. For those ASHAs that were less comfortable with this role, follow-up review by the doctor and confirmation that they had performed the risk assessment accurately were key elements that would increase confidence.

There were also accounts of role conflict with other senior NPHWs, in particular auxiliary nurse midwifes (ANMs). ANMs are in charge of village subcenters. They are more highly trained, receive higher remuneration, and carry broader responsibilities in delivery of PHC activities than ASHAs. Although most ASHAs worked well with ANMs, there were some accounts of hostility from the ANM:

...she (the ANM) scolded me and said that I am giving too much importance to this work...I said I am doing both jobs...and it is my problem...but I made 1 mistake...I regretted not showing this tablet to her...

ASHA 6, 32 years

Most ASHAs, however, felt that if the ANM was actively informed about the program and if existing work duties were not compromised then potential conflict with ANMs would be minimized.

#### Workforce Conditions and Entitlements: Diminished Opportunity and Motivation

Despite the untapped potential of the ASHA workforce to perform expanded roles, insufficient remuneration, lack of job security, and availability of other more lucrative employment opportunities in non–health-related areas constitute critical threats to sustainability of this workforce:

There were 4 ASHAs initially working in this village, but now 1 member has left to work in Kuwait and another member has left due to insufficient salary...It (the job) is good but the salary is insufficient.... Despite 6 years of experience it is still not permanent...

ASHA 1, 31 years

The departure of ASHAs from the village places additional pressures on the remaining ASHAs. Although the government targets are set at 1 ASHA per 1000 population, this is not always reached:

Earlier 3 ASHAs used to work here and now 2 have left...I used to look after 1000 people and now I have to look after all 2700 people.

ASHA 2, 30 years

### Broader Health System Barriers to Actual Health Care Transformation

#### Drug Access and Adherence: Diminished Opportunity and Motivation

Although the ASHAs recognized the importance of identification of high-risk individuals and confirmed the feasibility of incorporating this program in their daily schedule, there was some apprehension about the success of the program due to the availability of medications. ASHAs described a gap in care postreferral, where the patient would need to visit a doctor and, if required, would have to take medications. Private consultation costs (if any due to referral by the PHC doctor) and the availability of medicines at the PHC were the most prominent barriers identified:

...My doing testings for BP or sugar is quite acceptable to them. But, for all those ailments, there is a need to give medication. If medication was also supplied, it would become a full-fledged program.

ASHA 9, 33 years

Free medicines are available for the patients at the PHCs, but they need to be prescribed first by the doctor. Community participants suggested that dispensing of medicine in the village would greatly improve the service. Another potential implication was that by increasing access to more comprehensive screening there would be downstream pressures on the PHC physicians to treat ever-increasing numbers of people with limited medication supplies.

#### Doctor Access: Diminished Opportunity

Although the intervention strategy has great potential to overcome navigation barriers via ASHA-led household screening assessments, there were substantial “downstream” barriers uncovered in relation to access to doctors. ASHAs expressed the nonavailability of the medical officer at PHC during the patient’s visit and conflicting advice from the medical officer would lessen the confidence of the community in the ASHAs:

If we refer somebody and if the doctor examines them, then the patients would respect us and it will be good...if it is not done, there may be a problem. If the doctor won’t examine or is not available when we send patients, then we will lose impression.

ASHA 1, 31 years

One community participant described how she was recommended for referral for treatment of her elevated blood pressure. She went to the government PHC on 2 occasions with the expectation of commencing treatment; however, on both occasions there were no doctors present at the clinic. The lack of availability of doctor services and perceived hostility by nondoctor PHC staff leads community members to seek care in the private system. Large out-of-pocket costs can ensue as a consequence:

People are pawning household utensils to afford going to private clinics...Even if we go to the government hospital, their treatment of patients is bad...As long as the doctor is around, nurses behave, once the doctor is gone they do not care and all of them also go away...They sometimes ask us to go to other private hospitals...How can poor people afford that?

Female focus group 2

## Discussion

### Principal Findings

This field evaluation of a multifaceted mHealth strategy to improve CVD risk screening and management found that such a strategy can be successfully developed and integrated with the current tasks performed by ASHAs and physicians. Drawing on behavior change theory, the strategy showed promising signs of enhancing ASHA capability and motivation to perform village-based CVD risk screening and provided new opportunities for these workers to perform an expanded role. The strategy also appeared to increase opportunities for patients by increasing access to screening facilities within the home at times that are more convenient. Equally important, treatment gaps were also identified by the tool, which resulted in appropriate management by the doctors. The evaluation also highlighted important constraints once high-risk individuals were referred to the local PHC for treatment. Only one-third of patients referred visited a local physician and only approximately one-third of those who were advised medication by the physician were still taking medication after 3 months. Reasons for poor engagement with the health system are not clear, but might include poor accessibility to physicians, costs associated with travel to visit a doctor, remoteness of the place of stay, proper counseling about lifelong therapy with drugs, and out-of-pocket costs for these drugs [30]. These critical issues of access to care and use of medication are similar to other studies conducted in this setting [30-32]. Although the numbers are too small to make any definitive conclusions, a greater proportion of patients managed by the public system were adherent to recommended treatments at 3 months compared with those seen privately. This suggests that both provider and patient-level factors may manifest differently in the public versus private systems. There were also additional potential constraints related to insufficient remuneration and role conflict with other nonphysician health staff. Addressing these key barriers is likely to be essential for mHealth strategies to be successfully taken up in the Indian government primary health care sector.

The findings highlight that mHealth interventions cannot exert their effect in isolation of prevailing health system context. The Innovative Care for Chronic Conditions (ICCC) Framework [33] stresses that health care is a complex interplay of different actors, requiring end-to-end solutions that address constraints and enhance opportunities at multiple levels. Labrique and colleagues [34] conceptualized mHealth as a systems-strengthening tool integrated within the context of the broader health system. They described 12 common mHealth applications, including interventions focused on health behavior change, diagnosis, data collection and health records, health worker training, human resource management, supply chain management, and financial transaction. The authors recommend that mHealth projects deploy multiple intervention types to maximize success. When placed in this context, the SMARTHealth India strategy encompasses many, but not all, of these areas. An increased emphasis on human resource management, supply chain management, and financial transactions appear to be critical areas for expansion of the platform that will address some of the critical barriers highlighted in this study. Taking all these factors into account, 5 key actions have been identified to optimize the SMARTHealth India strategy:

1. CDSS tool enhancements: Simplification of the user interface, proper language font installation, and greater emphasis on the use of color for communicating risk to the patients are key technical issues that need addressing. Collaboration with local mobile phone operators to boost signal strength in villages with weak network connectivity could also greatly improve data use. There is also a need to ensure more accurate capture of medical history by asking questions in a more standardized fashion. The tablet provides an ideal vehicle for incorporation of multimedia resources to facilitate this. NPHWs could demonstrate short animations during their routine visits to obtain a more informed and consistent medical history (eg, regarding heart attack, stroke). This also provides an opportunity to provide a more interactive health education vehicle for patients.
2. Changes to the workflow of PHC staff to increase access: Potential strategies include engaging another intermediary work force (nurse, pharmacists) in the PHC other than the doctor, changes to the working arrangements of the doctors, and bringing the doctor to the village rather than the village to the doctor via structured community visits. Overcoming access blocks are likely to make a substantial impact on patient confidence and satisfaction with the PHC system and reduce financial impact associated with use of private practitioners.
3. Addressing drug supply chain barriers: Close engagement with local, district, and national government authorities is necessary to ensure that medicines on the national approved essential medicines list are reliably able to be accessed at the PHC level. Providing PHC medical officers and pharmacists with increased powers to indent these medicines and provision of mobile health applications to easily monitor critical resource shortages are potentially important solutions. As a result of this study, a closer collaboration with the ministry of health at the national, state, and district levels has been initiated and negotiations to ensure adequate CVD medication supply to the PHCs are currently underway. Future initiatives might incorporate prescription of a limited range of essential drugs by community-based NPHWs, similar to strategies widely adopted for the management of human immunodeficiency virus (HIV) infection [35].
4. Innovative strategies to improve treatment adherence: Addressing upstream system barriers to treatment adherence is important, but the sharp fall in persistence with treatments once initiated dramatically negates any benefits afforded by those treatments. Scalable strategies to increase knowledge, self-monitoring, repeated prompts, care coordination, and cost barriers are key factors that need addressing [36]. Although the evidence base is still immature, technological strategies such as text message systems have been shown to improve adherence in a variety of settings [37]. Attention to local context, however, is important. Most residents in Indian rural communities do not use text messages as a usual mode of communication and the most promising health strategies in this setting have been with the use of interactive voice messaging systems [38]. Voice messages can be used to support better use of medicines, treatment follow-up, and support with health behavior change such as smoking cessation. Voice systems can also be used with doctors and ASHAs to establish recall and reminder systems.
5. Remuneration and training and support for ASHAs: The detailed accounts of workforce constraints faced by ASHAs and doctors highlight the fact that human resource support must be incorporated as a core element to any strategy. Large-scale training of a community workforce to conduct household screening is not likely to be feasible under the workforce conditions experienced in this study. The expanded responsibilities associated with this role also have potential to divert the ASHAs from performing their usual maternal and child health duties. Addressing this issue also requires close collaboration with local, district, and national government authorities. Discussions are currently underway with the Indian government’s National Rural Health Mission to explore establishment of a dedicated noncommunicable disease ASHA workforce, drawing on the successful model utilized with maternal and child health programs. Based on these study findings, key factors to address in the establishment of such a workforce will include review of training requirements (including use of mHealth remote learning platforms), payment structures such as performance-based remuneration, improved management structures within the PHC, and integration rather than competition with other workforces such as ANWs.

This evaluation was highly exploratory in nature and examined a single screening encounter by the health worker and management by the physician. Rather than a representative sample, the study used convenience sampling whereby people residing in the villages were opportunistically selected based on the proximity or convenient accessibility to the physicians or ASHAs. Because the physician also screened patients from outside the 11 villages that were included in the study, it was not logistically possible to get follow-up information from these patients. It may be argued that general education and training for the ASHAs regardless of the technology could improve CVD risk screening, but it would almost inevitably need a more intensive formal training program in combination with continuous education to re-enforce the learned skills. The motivation for this study is to test an overall intervention strategy that aims to integrate health care between ASHAs and doctors. If successful, this could eventually be delivered at scale for a fraction of the cost of more traditional workforce strategies. The technology simplifies the process and facilitates the delivery of the information to the community through the health workers and physicians. A few ongoing trials are testing the difference in effectiveness using an electronic decision support system and a paper-based version of the tool [39]. These studies may shed light on the relative merits of computer- versus paper-based tools; however, it is clear that a paper-based tool lacks the systems-integrating element that a mHealth intervention can provide. The main focus of the study was to understand the acceptability and feasibility of the strategy in preparation for a more robust, large-scale evaluation. mHealth interventions are frequently criticized for being nonrepresentative in nature, dominated by pilot studies, and not focused on scalable solutions. Although this is certainly true, the critical insights learned from this exploratory study highlight the importance of detailed scoping work before undertaking large and expensive evaluations.

The findings reported in this study are likely to be relevant to other LMICs with similar health system contexts. The WHO Package of Essential Noncommunicable Disease Interventions [40] provides a comprehensive vehicle in which to take a systems approach to mHealth strategies. This package explicitly recommends investment in early disease detection using affordable technology, pharmacological and nonpharmacological management of risk factors, and provision of affordable medicines for treatment and prevention [40]. Such features, if successfully implemented at all levels of the health care system, have the potential to bring about transformative changes to health care access and quality. A key element underpinning the successful and scalable implementation of the WHO package is workforce transformation. Although there is well-established literature on the effectiveness of task-shifting the physician role to NPHWs in HIV/AIDS care settings [41], there have been few studies conducted to date that have focused on the transformative role of NPHWs in managing CVD, although some studies have shown evidence that it has helped in managing single risk factor management [42,43]. This study goes some way to outlining that similar success from task shifting for noncommunicable disease management may be possible, but only if viewed within a broader health system context.

### Conclusions

This feasibility study provides initial insights on the acceptability and preliminary effectiveness of a tablet-based DSS to improve CVD detection, prevention, and management in an Indian primary health care setting. It incorporates a technological solution with innovative workforce strategies to address the growing CVD epidemic in India. Despite great promise for mHealth interventions to improve access to effective health care, there remains considerable uncertainty about how this can be successfully achieved. Appreciation of the broader systems issues and integration of mHealth strategies within this broader context are essential factors in maximizing impact from such approaches. The intervention strategy will be optimized based on the recommendations from this study and it will be tested for clinical- and cost-effectiveness in a large randomized trial in a similar setting [44]. If found to be successful, the findings are likely to advance knowledge on scalable strategies to improve access to effective health care for underserved populations worldwide.

## Abbreviations

ANM: auxiliary nurse midwife
ASHA: accredited social health activist
BP: blood pressure
CDSS: clinical decision support system
CHD: coronary heart disease
CVD: cardiovascular disease
HDL-C: high-density lipoprotein cholesterol
LDL-C: low-density lipoprotein cholesterol
LMICs: low- and middle-income countries
mHealth: mobile health
NPHW: nonphysician health care worker
PHC: primary health care center
PVD: peripheral vascular disease
TC: total cholesterol
